# Impact of Brood Cell Cocoons on Metal Accumulation and CYP450 Detoxification Gene Expression in *Apis cerana cerana*

**DOI:** 10.3390/toxics12020131

**Published:** 2024-02-06

**Authors:** Qingxin Meng, Rong Huang, Shunhua Yang, Hui Li, Dan Yue, Xueyang Gong, Wenzheng Zhao, Yakai Tian, Kun Dong

**Affiliations:** Yunnan Provincial Engineering and Research Center for Sustainable Utilization of Honeybee Resources, Eastern Bee Research Institute, College of Animal Science and Technology, Yunnan Agricultural University, Kunming 650201, China; 13015572330@163.com (Q.M.); huangrongg2022@163.com (R.H.); fengxue_20141011@163.com (S.Y.); 13769308931@163.com (H.L.); yuedan2015@126.com (D.Y.); xueyangg11@126.com (X.G.); rurosezwz@163.com (W.Z.)

**Keywords:** cocoon, metals, CYP450 gene, old comb, comb-gnawing

## Abstract

Honey bees play a critical role as pollinators. However, their reproduction success and survival face severe threats due to the deterioration of their living environment. Notably, environmental conditions during their preimaginal stage inside brood cells can influence their immune capabilities and overall health after emergence. During the in-cell developmental stage, workers are in close contact with cocoons, which can become a source of stress due to accumulated metals. To investigate this potential threat, experiments were conducted to examine the impact of cocoons in brood cells used to rear different generations on the metal content and detoxification gene expression levels in *Apis cerana cerana*. Our findings indicated significant differences in the layers, weight, base thickness, and metal contents like Cr, Cd, Pb, Mn, Ni, and As of cocoons in multi-generation brood cells compared to single-generation brood cells. These increases led to significant elevations in metal levels and upregulations of the four CYP450 detoxification genes in both six-day-old larvae and newly emerged workers. In conclusion, this study highlights the negative impact of cocoons in multi-generation brood cells on bee health and provides evidence supporting the development of rational apiculture management strategies for ecosystem stability.

## 1. Introduction

Honey bees are a crucial part of terrestrial ecosystems and are essential for maintaining the diversity and stability of plant populations as the main pollinators of most crops and wild plants [1]. However, with significant colony declines in recent years, honey bees face serious threats to their survival [2]. In 2006–2007, colony collapse disorder (CCD) emerged in the United States and Australia, leading to colony losses of more than 50% by local beekeepers, and this phenomenon subsequently spread to other countries [3,4]. These colony losses have attracted widespread attention, prompting many scientists to investigate the underlying causes. Currently, potential biotic (e.g., pathogens, pests) and abiotic (e.g., climate change, habitat loss) factors contributing to colony declines have been extensively studied [5,6,7]. However, the exact causes have not yet been thoroughly investigated, and the search for potential factors remains urgent.

In recent scientific studies, the drawbacks of old combs on colonies have gained attention. Specifically, old combs produce a positive feedback regulation that leads to several negative effects on the colony [8,9]. Compared to newly built or fresh combs, the structure of old brood cells is altered, leading to a volume reduction of about 9% [10,11]. This results in workers with lighter birth weights and reduced external morphometric sizes [12]. It also decreases both the lifespan of workers and the colony population [13]. Importantly, these changes negatively impact the colony strength and productivity [14]. Generally speaking, the volume reduction in old brood cells, caused by the accumulation of cocoons and other materials produced by broods during the in-cell developmental stage, is a key factor causing these effects [15]. The cocoon, composed of honey bee silk secreted during the spinning larval stage, adheres to the cell wall along with feces and other secretions [16]. With the habit of reusing cells for brood-rearing, the layers and quality of the accumulated cocoon increase with each new worker’s generation [11]. Thus, the temporal (through multiple generations) and spatial (occupy cell volume) accumulation characteristics of cocoons in the brood cells may have the potential to continuously accumulate harmful substances such as heavy metals.

Heavy metals are naturally occurring metals with an elemental density greater than 5 g/cm^3^ and an atomic number above 20 [17]. They primarily originate from agricultural practices, metallurgical activities, energy production, transportation, and microelectronic waste disposal [18]. In particular, metals can enter and accumulate in the food chain, which is harmful to human health, leading to a series of disorders including neurotoxicity, immune problems, and carcinogenicity [19,20]. Metal exposure also disrupts honey bees’ cognition (learning, memory, and olfaction), detoxification, and immunity, threatening their foraging behavior and lifespan [21,22,23]. With industrialization and urbanization, the concentration of metals in the natural environment has increased significantly [24]. Plants in metal-rich soils can absorb and accumulate these metals, transferring them to their aboveground parts and secretions like nectar and pollen [25]. Foraging bees can carry metals from contaminated sources into the hive to produce bee products including honey and beebread, etc. [26]. As the container for these products, beeswax, which is primarily composed of lipophilic substances, inevitably accumulates metals [27]. Moreover, metals can bioaccumulate inside the body of the bees through the ingestion of food or water and can then be transferred to the larvae by the feeding activity [28,29]. Due to their close contact, the metals in these matrices may migrate and accumulate in the cocoon with the increase in bee generation.

The early developmental environment significantly impacts honey bees’ immune capabilities, social behaviors, learning, and memory [30,31]. Especially, honey bees undergo complete metamorphosis, with the egg, larva, and pupa stages all occurring within the brood cell [32]. This ensures that each worker is in close contact with the cocoon for at least 3 weeks, while the lifespan of workers after emergence is typically 3–6 weeks during the spring and summer [16,33]. However, it is unclear whether the health of workers developed in old brood cells is affected by increased metals hoarded in generation-accumulated cocoons. Metals like chromium (Cr), cadmium (Cd), lead (Pb), manganese (Mn), nickel (Ni), arsenic (As), and mercury (Hg) have been studied to possess significant toxic effects on bees [34,35,36,37,38]. Content surveillance of these metals in the hive matrix can lead to a greater understanding of the environmental threats to bees. Notably, the CYP450 gene family, which is considered an indicator of pollutant presence and insect health, plays a significant role in the tolerance of honey bees to environmental stress [22]. Therefore, this study focused on *Apis cerana cerana* (Fabricius, 1793) to investigate the impact of cocoons within brood cells on the metal content and detoxification gene expression levels in workers. Our research includes: (1) measuring structural characteristics of cocoons in single-generation and multi-generation brood comb cells; (2) detecting the content of seven metals in cocoons and in six-day-old larvae and newly emerged workers reared in them; and (3) analyzing detoxification gene expression levels in workers reared in both brood cell types, revealing the negative impact of accumulated cocoons in brood cells on the health state of workers. Overall, this study provides a basis for formulating beekeeping management strategies for *A. c. cerana*, aiming to protect honey bee survival and ecosystem stability. 

## 2. Materials and Methods

### 2.1. Information on the Sampling Site

**Figure 1:**
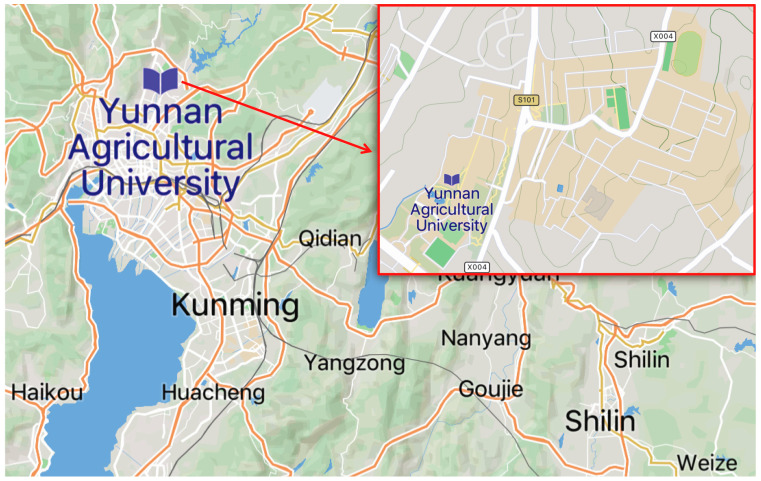
Sampling site in Kunming, Yunnan, China. The nest icon in the upper right corner represents the location of the experimental apiary. This map was downloaded from https://mapcarta.com/W302022003 (accessed on 20 December 2023).

This study was conducted at the experimental apiary of the Eastern Bee Research Institute at Yunnan Agricultural University (Kunming, Yunnan, China, 25°13′ N, 102°75′ E, altitude 1931 m). The apiary is surrounded by several major transportation routes with substantial automobiles. Additionally, there is an industrial park engaged in mechanical processing production southeast of the apiary (Figure 1).

### 2.2. Colonies Establishment and Test Sample Collection

**Figure 2:**
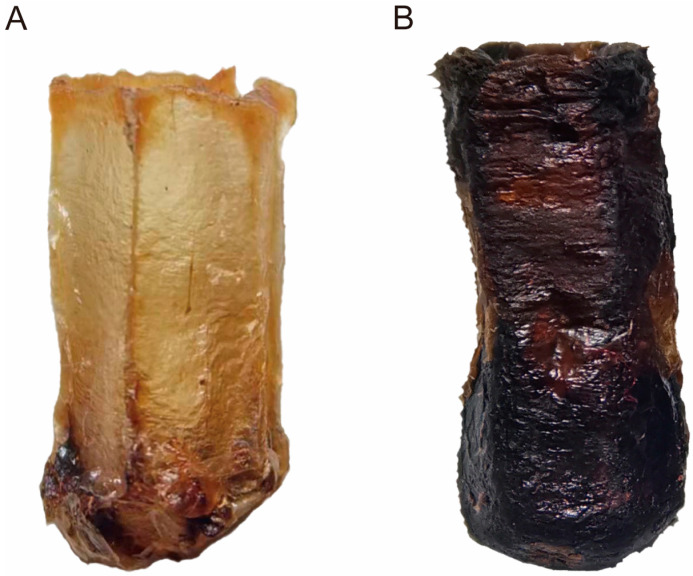
Accumulated cocoons inside (**A**) single-generation combs reared for one generation of workers and (**B**) multi-generation combs reared for numerous generations of workers, with gnawed marks in the brood-rearing area and used for one year.

Initially, two types of experimental combs were collected. These included three single-generation combs that had reared one generation of workers and three multi-generation combs that had reared numerous generations of workers (approximately eight generations), with a 1-year age and with some brood cells gnawed by workers [11,39]. Then, a 50 cm^2^ comb block was cut from each comb’s brood-rearing area using an uncapping knife. It was placed in a solar wax melter to isolate the cocoons by removing the beeswax, and 60 cocoons were collected from each comb (Figure 2). Subsequently, six colonies of *A. c. cerana* were established in standard Langstroth ten-frame hives, each starting with a strength of six full frames. Three of the colonies were provided a single-generation comb, while the other three colonies received a multi-generation comb. Moreover, the remaining five combs in all colonies were replaced with newly built combs. Finally, the queens of each colony were placed in oviposition controllers with the experimental combs, and the dates of oviposition were recorded. On the ninth day, 30 six-day-old larvae were collected from each comb. As workers were about to emerge, each comb was placed in an incubator with controlled temperature (35 ± 0.1 °C) and humidity (75 ± 0.1%). From each comb, 30 newly emerged workers were collected.

### 2.3. Measurement of Cocoon Structural Characteristics

For each experimental comb, 10 cocoons were randomly selected for paraffin section preparation. The cocoons were first fixed with 4% paraformaldehyde. They were then cut along the longitudinal axis, embedded using a paraffin embedding station (Leica, Wetzlar, Germany, HistoCore Arcadia H), and sliced into 4 µm thick sections using a rotary microtome (Leica, Germany, HistoCore BloCu7). Additionally, the samples were placed in cold water, then placed in 45 °C water for spreading, and then attached to glass slides heated to 70 °C in an oven for 1–2 h. Finally, hematoxylin and eosin (H&E) staining was performed, followed by brightfield scanning to obtain cocoon paraffin sections. 

Furthermore, 20 cocoons from each comb were selected for weight and base thickness measurement. The cocoon weight was measured using an electronic balance. They were then cut longitudinally with anatomical scissors, and the base thickness was measured using a CCD video microscope (OLYMPUS BX53, Suzhou Jing Kai instrument and Equipment, Suzhou, China).

### 2.4. Metal Content Analysis: Sample Preparation and Measurement

The contents of seven metals (wet weight), including chromium (Cr), cadmium (Cd), lead (Pb), manganese (Mn), nickel (Ni), arsenic (As), and mercury (Hg), were detected in the accumulated cocoons, six-day-old larvae, and newly emerged workers from both single-generation and multi-generation comb cells, and 30 samples of each type were collected from each experimental comb. Specifically, arsenic is considered a metal even if it is a metalloid. Initially, 5 mL of a 100 µg/mL mixed calibration standard containing 28 metals (National Center of Analysis and Testing for Nonferrous Metals and Electronic Materials, Beijing, China) was measured into a 100 mL volumetric flask. This was diluted with 1% nitric acid (Aladdin, Shanghai, China) and mixed to prepare a 5 mg/L intermediate solution. Then, a 100 μg/mL internal standard solution of six metals (National Center of Analysis and Testing for Nonferrous Metals and Electronic Materials, Beijing, China) was serially diluted with 1% nitric acid to 50 μg/L. Subsequently, 0.1–0.5 g (accurate to 0.0001 g) of the sample was weighed into a microwave digestion tank (Preekem Scientific Instruments, Shanghai, China). To this, 5 mL of nitric acid was added and predigested on a graphite digestion apparatus (Zhejiang Tuojie Instrument, Yiwu, China) at low temperatures. When the yellow smoke was exhausted, digestion was performed according to the standard operation steps. The temperature was linearly increased stepwise, reaching 120 °C for 5 min, then 160 °C for 5 min, and finally 185 °C for 25 min. After heating, when the vessels were cooled down to room temperature, they were placed on an acid extractor (Hanon Advanced Technology Group, Jinan, China) to remove the acid and dried. Then, they were adjusted to a volume of 10 mL with 1% nitric acid and mixed. Concurrently, a blank sample was prepared. Additionally, an iCAP TQ ICP-MS (ThermoFisher, Waltham, MA, USA) was used for the detection of the metal content. The main operating parameters and acquisition conditions of the instrument were as follows: tuning mode STD/KED; dwell time: 0.1 s; sample introduction speed and time: 40 rpm, and 40 s; plasma power: 1550 W; sampling depth: 5.0 mm; nebulizer gas flow: 0.98 L/min; cooling gas flow: 14.0 L/min; auxiliary gas flow: 0.8 L/min; nebulizer chamber temperature: 2.7 °C; torch horizontal and vertical position: 0.16, and 0.53 mm; He flow: 4.55 mL/min; O_2_ flow: 0.3125 mL/min; lens D1 voltage: 350 V; lens D2 voltage: 154 V; number of replicates: 3 times. All samples underwent triplicate analyses, and the recovery values (%), limits of detection (LOD, mg/kg), and limits of quantification (LOQ, mg/kg) for each metal were as follows: Cr (99.7, 0.0052, and 0.0208), Cd (101.3, 0.0039, and 0.0156), Pb (96.3, 0.0040, and 0.0160), Mn (98.6, 0.0103, and 0.0412), Ni (98.5, 0.0090, and 0.0360), As (98.5, 0.0052, and 0.0208), and Hg (94.4, 0.0123, and 0.0492). Among them, the content of Hg fell below the LOD and LOQ. The calculation formula was as follows: metal content = (C − C0) × 10 × Dilution factor/Sampling amount/1000. Content unit: mg/kg; C (concentration of metal ions in the detection sample) unit: μg/L; C0 (concentration of metal ions in the blank sample) unit: μg/L; sampling unit: g.

### 2.5. Total RNA Extraction, cDNA Synthesis, and RT-qPCR Analysis

According to the manufacturer’s protocol, TRNzol Universal Reagent (TIANGEN, Beijing, China) was used for extracting total RNA from the *A. c. cerana*. Firstly, TRNzol Universal was added and shaken after grinding the honey bees in a centrifuge tube. After that, chloroform was added and mixed, and the RNA was dissolved in the supernatant by centrifugation using a refrigerated centrifuge (FUP A17CH, Qingdao Yizhuo Photoelectronic Technology, Qingdao, China) at 12,000 r/min at 4 °C. The supernatant was then transferred to another tube and mixed with isopropanol. The RNA precipitate, obtained by centrifuging again, was washed with 75% ethanol and allowed to dry. Finally, it was dissolved by adding RNase-free H_2_O to obtain the total RNA from the honey bees. First-strand cDNA was synthesized using a FastKing RT Kit (TIANGEN, Beijing, China). The A260/A280 value of all samples was determined using a micro-volume nucleic acid spectrophotometer (HM-CWF1, Shandong hengmei electronic technology, Weifang, China). Furthermore, *β-actin* was used as the reference gene. *AccCYP4AV1*, *AccCYP314A1*, *AccCYP4AZ1*, and *AccCYP6AS5* were selected as the target genes. The primer sequences for each gene are listed in Table 1. In addition, a 10 μL reaction mixture was prepared according to the SuperReal PreMix Plus (TIANGEN, Beijing, China). The reaction conditions were as follows: pre-denaturation at 95 °C for 3 min, followed by 45 amplification cycles (denaturation at 95 °C for 15 s, annealing/extension at 60 °C for 30 s). The melt cycle ranged from 65 °C to 95 °C. The relative expression levels of each gene were calculated using the 2^−∆∆CT^ method.

### 2.6. Statistical Analysis

Statistical analysis was performed using the GraphPad Prism 9.5 software (GraphPad Software, Boston, USA). The normality of the experimental data for each group was tested using the Kolmogorov–Smirnov test, and all data were tested to show a normal distribution. T-tests were used to compare the differences in weight, base thickness, and metal content of the cocoon from single-generation and multi-generation comb cells. Moreover, T-tests were used to compare the differences in metal contents and gene expression levels in six-day-old larvae and newly emerged workers reared in two types of comb cells. Pearson’s correlation analysis was performed to find correlations between the cocoon structure characteristics, metal content, and detoxification gene expression level. The significance level was set at *α* = 0.05. Data are presented as mean ± standard error, where the standard error decreases as the sample size increases and the experimental sample becomes more representative of the whole.

## 3. Results

### 3.1. Paraffin Sections of the Accumulated Cocoons in Comb Cells

**Figure 3:**
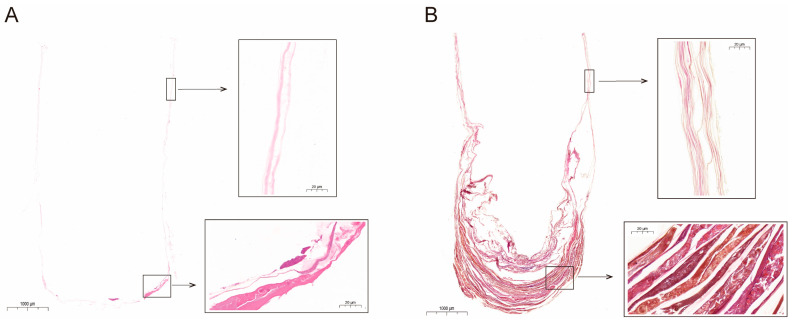
Paraffin section of the accumulated cocoons in (**A**) single-generation and (**B**) multi-generation comb cells.

In the single-generation comb cells, the cocoon wall was of a single layer. However, at the base, the number of layers was increased, with the accumulation of substances occurring (Figure 3A). Conversely, in the multi-generation comb cells, the cocoon wall was multilayered. Here too, the number of layers and substance accumulation at the base were further increased (Figure 3B).

### 3.2. Comparison of Cocoon Weight and Base Thickness

**Figure 4:**
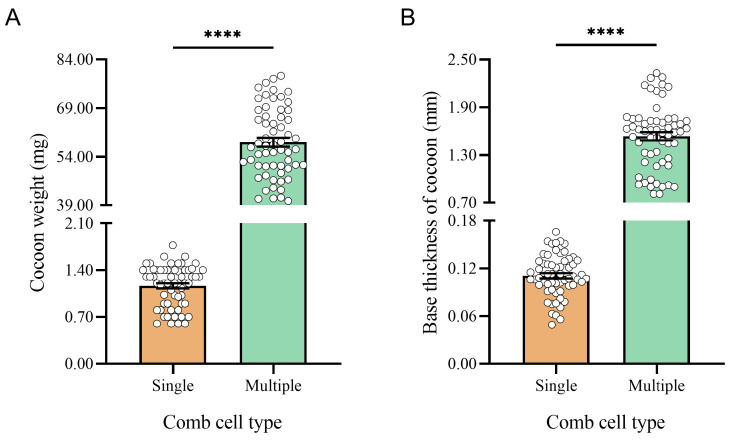
Comparison of (**A**) weight and (**B**) base thickness of cocoons in the single-generation and multi-generation comb cells. ****: *p* < 0.0001.

The cocoons in the multi-generation comb cells (58.48 ± 1.38 mg) weighed significantly higher on average compared to those in the single-generation comb cells (1.16 ± 0.04 mg) (t = 41.51; df = 118; *p* < 0.0001) (Figure 4A). Similarly, the average base thickness of the cocoons in the multi-generation comb cells (1.54 ± 0.05 mm) was significantly greater than those in the single-generation comb cells (0.11 ± 0.01 mm) (t = 27.32; df = 118; *p* < 0.0001) (Figure 4B).

### 3.3. Comparison of Metal Contents in Cocoons and Workers

#### 3.3.1. Comparison of Metal Contents in Accumulated Cocoons from Comb Cells

The cocoons from the multi-generation comb cells had significantly higher metal contents (Cr, Cd, Pb, Mn, Ni, and As; wet weight) compared to those from the single-generation comb cells (*p* < 0.05). However, Hg was not detected in any cocoon samples in this study (Table 2).

#### 3.3.2. Comparison of Metal Contents in Six-Day-Old Larvae

The larvae reared in the multi-generation comb cells contained significantly higher levels of metals (Cr, Cd, Pb, Mn, Ni, and As; wet weight) compared to those reared in the single-generation comb cells (*p* < 0.05). Identically, Hg was not detected in the larval samples from both comb cell types (Table 3).

#### 3.3.3. Comparison of Metal Contents in Newly Emerged Workers

The newly emerged workers from the multi-generation comb cells exhibited significantly higher metal levels (Cr, Cd, Pb, Mn, Ni, and As; wet weight) than those from the single-generation comb cells (*p* < 0.05). Similarly, Hg was not detected in the worker samples from either comb cell type (Table 4).

### 3.4. Comparison of the Expression Levels of CYP450 Genes in Workers

**Figure 5:**
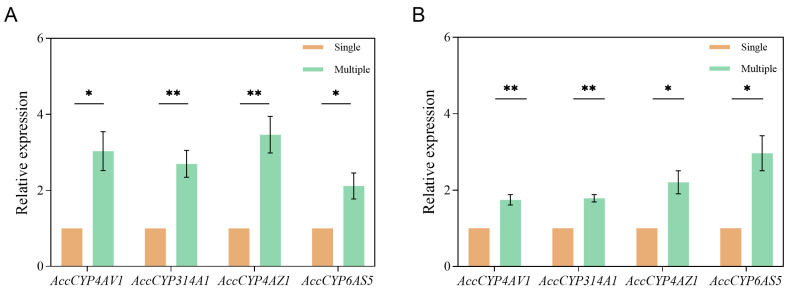
Comparison of expression levels of four CYP450 detoxification genes in (**A**) six-day-old larvae and (**B**) newly emerged workers reared in single-generation and multi-generation comb cells. *: *p* < 0.05, **: *p* < 0.01.

We found significant differences in the expression levels of four CYP450 genes in the six-day-old larvae reared in the single-generation and multi-generation comb cells (*p* < 0.05). The expression levels of *AccCYP4AV1* (t = 3.983, df = 4, *p* = 0.0164), *AccCYP314A1* (t = 4.799, df = 4, *p* = 0.0087), *AccCYP4AZ1* (t = 5.136, df = 4, *p* = 0.0068), and *AccCYP6AS5* (t = 3.272, df = 4, *p* = 0.0307) were significantly higher in the larvae from the multi-generation comb cells than those from the single-generation comb cells (Figure 5A).

Similarly, significant differences were found in the expression levels of these genes in the newly emerged workers reared in the single-generation and multi-generation comb cells (*p* < 0.05). The expression levels of *AccCYP4AV1* (t = 5.496, df = 4, *p* = 0.0053), *AccCYP314A1* (t = 8.180, df = 4, *p* = 0.0012), *AccCYP4AZ1* (t = 3.990, df = 4, *p* = 0.0163), and *AccCYP6AS5* (t = 4.289, df = 4, *p* = 0.0128) were significantly higher in the workers from the multi-generation comb cells compared to those from the single-generation comb cells (Figure 5B).

### 3.5. Correlation Analysis between Cocoon Structure Characteristics, Metal Content, and Detoxification Gene Expression Level

**Figure 6:**
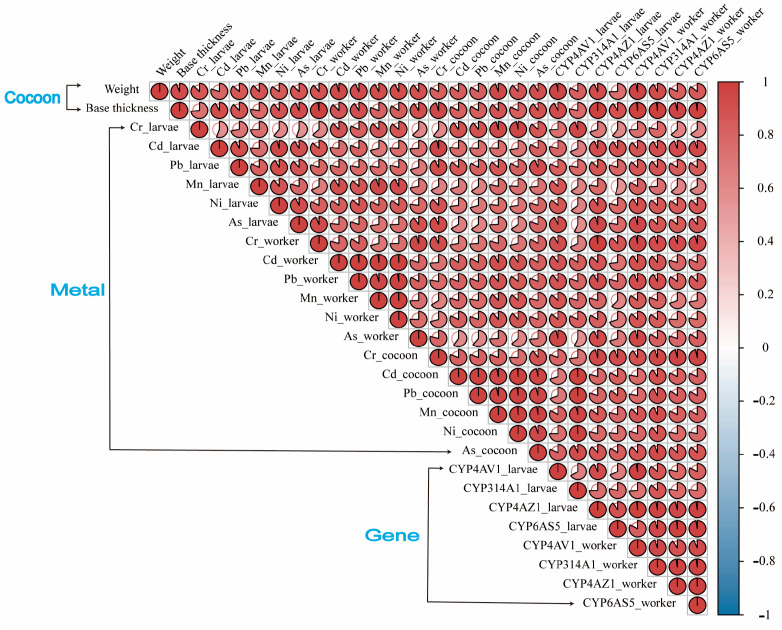
Correlation analysis among cocoon structure characteristics, metal content, and detoxification gene expression in *A. c. cerana*. Different shades of red indicate a positive correlation, where the size of the pie chart represents the magnitude of the correlation between the two indicators.

We found a significant positive correlation between the characteristics of cocoon structure (weight, base thickness), metal content, and the expression levels of detoxification genes in *A. c. cerana* (*p* < 0.05; Figure 6). This finding indicates that the accumulation of cocoons with a greater weight and base thickness and a higher metal content within the multi-generation comb cells corresponds to increased levels of metal content and an upregulated expression of CYP450 detoxification genes in the workers of *A. c. cerana* reared in these cells.

## 4. Discussion

### 4.1. Structural Characterization of the Cocoon and its Enrichment of Metals

The cocoon is composed of bee silk, feces, and other secretions released by the brood during the pre-pupal period [16]. Approximately 12–18 h after this period begins, the septum between the larva’s midgut and hindgut ruptures, and the larva subsequently excretes light brown or dark brown feces on the cell base [40]. In this study, examining the cocoon’s paraffin sections revealed a single-layer wall structure built by workers. It thickens at the base and accumulates feces. As the same brood cell can rear numerous generations of workers, the layers, weight, and base thickness of the cocoons significantly increase in multi-generation comb cells, leading to a reduction in cell volume [11]. However, the accumulated cocoons in the cell not only limits the developmental space of the brood, affecting the external morphology of the workers and negatively impacting the colony, but it also enriches metals [10,14,39]. Borsuk et al. (2021) discovered that metals can be immobilized or metabolized in the fat bodies of Western honey bees and then expelled as feces [41]. Our study indicates that metal concentrations, including Cr, Cd, Pb, Mn, Ni, and As, were significantly higher in the cocoons from the multi-generation comb cells. They increased by 1.59, 2.17, 2.12, 2.51, 2.55, and 1.7 times, respectively, compared to the single-generation comb cells (Table S1). Hence, the accumulation of feces in cells from multiple generations of workers indeed leads to increased metal content in the cocoon. Furthermore, *A. c. cerana* has a prominent biological behavior known as comb-gnawing, which is currently believed to be primarily caused by old combs resulting from rearing multiple generations of workers, with a reduced volume of brood cells and a not-well-developed morphological character of newly emerged workers [39]. The multi-generation combs used in this study showed comb-gnawing signs in the brood-rearing area. These surrounding brood cells, which were not yet gnawed and could still rear workers, were identified as the next ones to be gnawed [11]. Analyzing the metal content in the cocoons of these cells may help us further understand the potential factors like metal stress influencing the comb-gnawing behavior of *A. c. cerana*. In particular, finding over 100 mg/kg of Mn in the cocoon from the multi-generation comb cells and its content being increased by more than 2.5 times compared with the cocoon from the single-generation comb cells suggests that *A. c. cerana* might detect high Mn levels, potentially causing them to gnaw on old combs. However, further experimentation is necessary to confirm this. 

### 4.2. Metal Enrichment in Workers of A. c. cerana

Honey bees are important pollinators, but their survival and reproduction face severe threats due to deteriorating living conditions [2]. In particular, environmental conditions during the preimaginal stage inside the cell impact post-emergence immune ability and survival [31]. During the in-cell developmental stage, honey bees develop in direct contact with the cocoon, thus inevitably being affected by it. The result of the comparison of the ratios of the metal contents between the cocoons, larvae, and workers for the single or multi-generation comb cells showed that the cocoons were higher (Table S2). Furthermore, our research results show that the content of metals, such as Cr, Cd, Pb, Mn, Ni, and As, in larvae and adult workers cultivated in multi-generation comb cells is significantly higher than in single-generation comb cells. These metals have neurotoxic effects on honey bees and can damage their cognitive abilities and immune system functions [42]. Cognitive ability is crucial for honey bees’ adaptation to nature and colony life, encompassing learning, memory, olfaction, and gustation [43]. An assessment of exposure to field concentrations of metals (Pb and As) on the cognitive ability of honey bees revealed that bees in metal treatment groups had significantly reduced learning speed and memory abilities [21]. Similarly, exposure to Cd also leads to a decline in such abilities of honey bees [44]. The immune system, which is crucial for surveillance, defense, and regulation, can be compromised by exposure to polluted environments [45]. Pollutants can directly or indirectly affect the immune system of honey bees, increasing their susceptibility to diseases and harming their health [46]. A study found that Cd stress causes severe cellular damage in the fat bodies of honey bees, leading to a decline in immune capabilities [47]. In addition, trace essential elements play a vital role in the physiological metabolism of honey bees [42]. They are key factors in regulating hemolymph and tissue fluid osmotic pressure, promoting endocrine activity, and enhancing immune function. Among these, Mn acts as an activator for many enzymes in honey bees [48]. However, honey bees cannot perceive the metal content in the field environment, preventing them from effectively avoiding harm from metals [49]. Excessive elements in their habitat can also significantly harm honey bees [50].

### 4.3. Expression of CYP450 Detoxification Genes in Workers of A. c. cerana

Due to the rapid development of industry, insects have been exposed to environmental pollutants caused by human activities for an extended time [51]. Under environmental stress, the level of oxidative damage in organisms increases, leading to oxidative stress and the production of numerous reactive oxygen species (ROS) [2]. To resist the effects of environmental pollutants, honey bees have evolved with a variety of enzyme gene families for metabolic detoxification, including cytochrome P450 monooxygenases (P450s), which are responsible for synthesizing and degrading endogenous metabolites and detoxifying exogenous compounds, such as metals [52]. Due to genetic diversity and broad substrate specificity, the CYP 6 and CYP 9 clades within the CYP450 gene family are significant in enhancing insect tolerance to environmental stresses [53]. They are considered biomarkers of pollutant presence, with roles in metabolizing and detoxifying metals and other substances [54]. Zhang et al. isolated three CYP450 genes from the Chinese honey bee: *AccCYP314A1*, *AccCYP4AZ1*, and *AccCYP6AS5* [55]. When Chinese honey bees were exposed to the metals Cd and Hg, the expression of these genes increased significantly. However, when these genes were silenced using RNA interference technology and the honey bees were re-exposed to metals, the bees’ mortality rate increased. This indicated the critical role of these genes in detoxifying and resisting metal-induced toxicity in honey bees. Similarly, our study showed that the concentration of metals in cocoons from the multi-generation comb cells was higher than that in the single-generation comb cells. This led to an increased content of metals and the expression of four CYP450 detoxification genes in the bees developed in the multi-generation comb cells, implying that the workers were under metal stress. It also highlights the role of CYP450 genes in detoxifying metals in honey bees.

## 5. Conclusions

After comb cells rear multiple generations of workers, the layers, weight, base thickness, and the content of metals all increase significantly in the accumulated cocoons compared to cells rearing one generation. This accumulation also leads to an increased metal content in the workers reared in these multi-generation comb cells, as well as an elevated expression of detoxification genes. Thus, due to the metal-enriching capacity of cocoons and the potential risk to honey bee health, replacing old combs timely is crucial to avoiding the adverse effects of the cocoons on honey bees in beekeeping practices.

## Figures and Tables

**Table 1:** Gene information and primer sequences.

Gene		Primer Sequence (5′-3′)
Target gene	*AccCYP4AV1*	F: GTGTGGATGTCCTTCGATCC
		R: TTCCACTCGGCTTTTCTTTC
	*AccCYP314A1*	F: CTTCGGGTAGCTCTCACGTC
		R: ACTTTGTATCCGACCCGTTG
	*AccCYP4AZ1*	F: TTGGCCGAATCCAAATAAGT
		R: GCAAATCGTTGTCCAATGC
	*AccCYP6AS5*	F: AATGGGCAGAGAAGTGTTCG
		R: AAAGAATGGTGCGAATGTCC
Reference gene	*β-actin*	F: TTATATGCCAACACTGTCCTTT
		R: AGAATTGATCCACCAATCCA

**Table 2:** Metal contents in accumulated cocoons (wet weight) from single-generation and multi-generation comb cells (Mean ± S.E., unit: mg/kg).

Metals	Cocoons		Statistical Value	*p* Value
	Single	Multiple		
Cr	0.273 ± 0.024 ^a^	0.435 ± 0.038 ^b^	t = 3.577, df = 4	*p* = 0.0232
Cd	0.140 ± 0.012 ^a^	0.305 ± 0.028 ^b^	t = 5.432, df = 4	*p* = 0.0056
Pb	0.534 ± 0.024 ^a^	1.131 ± 0.105 ^b^	t = 5.517, df = 4	*p* = 0.0053
Mn	50.039 ± 2.134 ^a^	125.773 ± 6.333 ^b^	t = 11.320, df = 4	*p* = 0.0003
Ni	1.510 ± 0.092 ^a^	3.860 ± 0.358 ^b^	t = 6.358, df = 4	*p* = 0.0031
As	0.045 ± 0.005 ^a^	0.080 ± 0.002 ^b^	t = 6.923, df = 4	*p* = 0.0023
Hg	ND	ND	/	/

Note: different letters in the upper right corner of the same row indicate significant differences (*p* < 0.05), while “ND” denotes that the element was not detected by the method used in this study.

**Table 3:** Metal contents in six-day-old larvae of *A. c. cerana* (wet weight) reared in single-generation and multi-generation comb cells (Mean ± S.E., unit: mg/kg).

Metals	Six-Day-Old Larvae		Statistical Value	*p* Value
	Single	Multiple		
Cr	0.009 ± 0.002 ^a^	0.049 ± 0.009 ^b^	t = 4.257, df = 4	*p* = 0.0131
Cd	0.008 ± 0.001 ^a^	0.016 ± 0.002 ^b^	t = 3.385, df = 4	*p* = 0.0277
Pb	0.033 ± 0.006 ^a^	0.058 ± 0.002 ^b^	t = 3.879, df = 4	*p* = 0.0179
Mn	9.420 ± 1.132 ^a^	13.623 ± 1.014 ^b^	t = 2.777, df = 4	*p* = 0.0499
Ni	0.168 ± 0.042 ^a^	0.337 ± 0.037 ^b^	t = 3.054, df = 4	*p* = 0.0379
As	0.005 ± 0.001 ^a^	0.015 ± 0.003 ^b^	t = 2.991, df = 4	*p* = 0.0403
Hg	ND	ND	/	/

Note: different letters in the upper right corner of the same row indicate significant differences (*p* < 0.05), while “ND” indicates that the element was not detected.

**Table 4:** Metals contents in newly emerged workers of *A. c. cerana* (wet weight) reared in single-generation and multi-generation comb cells (Mean ± S.E., unit: mg/kg).

Metals	Newly Emerged Workers		Statistical Value	*p* Value
	Single	Multiple		
Cr	0.046 ± 0.004 ^a^	0.084 ± 0.009 ^b^	t = 3.825, df = 4	*p* = 0.0187
Cd	0.005 ± 0.003 ^a^	0.022 ± 0.002 ^b^	t = 5.565, df = 4	*p* = 0.0051
Pb	0.070 ± 0.022 ^a^	0.195 ± 0.009 ^b^	t = 5.228, df = 4	*p* = 0.0064
Mn	5.803 ± 1.587 ^a^	13.963 ± 1.284 ^b^	t = 4.000, df = 4	*p* = 0.0161
Ni	0.068 ± 0.040 ^a^	0.271 ± 0.019 ^b^	t = 4.581, df = 4	*p* = 0.0102
As	0.034 ± 0.002 ^a^	0.049 ± 0.005 ^b^	t = 2.966, df = 4	*p* = 0.0413
Hg	ND	ND	/	/

Note: different letters in the upper right corner of the same row indicate significant differences (*p* < 0.05), while “ND” indicates that the element was not detected.

## Data Availability

Data are available upon request to the corresponding author.

## Supplementary Materials

The following supporting information can be downloaded at: https://www.mdpi.com/article/10.3390/toxics12020131/s1, Table S1: Ratios of metal contents (wet weight) in the three matrices (cocoons, six-day-old larvae, and newly emerged workers) between the single- and multi-generation comb cells (unit: %); Table S2: Ratios of metal contents (wet weight) between the cocoons, six-day-old larvae, and newly emerged workers for the single- or multi-generation comb cells (unit: %).
