# Supplementary material for: Impact of Brood Cell Cocoons on Metal Accumulation and CYP450 Detoxification Gene Expression in Apis cerana cerana
Source: Toxics. 2024 Feb 6;12(2):131. doi: 10.3390/toxics12020131 (PMC10892446; doi:10.3390/toxics12020131)
Supplement: Supplementary file 1 [file toxics-12-00131-s001.zip › toxics-2827590-supplementary.pdf]

Table S1. Ratios of metal contents (wet weight) in the three matrices (cocoons, six-day-old larvae, and newly emerged workers) between the single and multi-generation comb cells (unit: %).

Metals	Cocoons		Larvae		Workers	
	Single	Multiple	Single	Multiple	Single	Multiple
Cr	38.54	61.46	15.73	84.27	35.22	64.78
Cd	31.44	68.56	32.39	67.61	18.60	81.40
Pb	32.06	67.94	36.54	63.46	26.55	73.45
Mn	28.46	71.54	40.88	59.12	29.36	70.64
Ni	28.12	71.88	33.25	66.75	19.98	80.02
As	35.91	64.09	26.40	73.60	41.33	58.67
Hg	/	/	/	/	/	/

Table S2. Ratios of metal contents (wet weight) between the cocoons, six-day-old larvae, and newly emerged workers for the single or multi-generation comb cells (unit: %).

Metals	Single			Multiple		
	**Cocoons**	**Larvae**	**Workers**	**Cocoons**	**Larvae**	**Workers**
Cr	83.26	2.81	13.93	76.55	8.67	14.78
Cd	91.67	5.07	3.26	88.95	4.71	6.34
Pb	83.76	5.20	11.05	81.77	4.16	14.07
Mn	76.67	14.43	8.89	82.01	8.88	9.10
Ni	86.49	9.62	3.88	86.38	7.55	6.07
As	52.92	6.26	40.82	55.61	10.27	34.11
Hg	/	/	/	/	/	/
